# Sepsis and disseminated intravascular coagulation are rare complications of typhoid fever: A case report

**DOI:** 10.1016/j.amsu.2021.103226

**Published:** 2022-01-03

**Authors:** Nurnaningsih, Vincencius William, Desy Rusmawatiningtyas, Firdian Makrufardi, Intan Fatah Kumara

**Affiliations:** Department of Child Health, Faculty of Medicine, Public Health and Nursing, Universitas Gadjah Mada/Dr. Sardjito Hospital, Yogyakarta, 55281, Indonesia

**Keywords:** Typhoid fever, Disseminated intravascular coagulation, Sepsis, Pediatric, Case report

## Abstract

**Introduction:**

and importance: Typhoid fever is an infection caused by *Salmonella typhi*. The common complications are intestinal perforation and typhoid encephalopathy. Cases of typhoid fever with sepsis and/or disseminated intravascular coagulation (DIC) are rarely reported, even though typhoid fever is endemic in Indonesia.

**Case presentation:**

A 4-year-old male referral case from a district hospital was experiencing fever, decrease of consciousness and massive bleeding from his gastrointestinal tract and nose. Investigation revealed results from the IgM typhoid test using Tubex®TF, with the score of +8. PELOD 2 score was 10, and PSOFA was 5. DIC score was 7. Based on these findings, the patient was diagnosed with typhoid fever, with DIC and sepsis being the complication of the typhoid fever.

**Clinical discussion:**

DIC is mostly a subclinical event, and severe bleeding complications found in typhoid fever are uncommon, although DIC scores which indicate an imbalance of coagulation and fibrinolysis are markedly elevated in patients with typhoid. DIC can be a part of multi-organ dysfunction due to sepsis syndrome. Acute infection can also result in systemic activation of coagulation.

**Conclusion:**

Sepsis and DIC are rare complications of typhoid fever. Typhoid fever can be presented with profound bleeding manifestation other than gastrointestinal bleeding, since it is a common symptom of typhoid fever. Further research should be conducted to postulate association between typhoid fever and DIC.

## Highlights

• Sepsis and DIC are rare complications of typhoid fever.
• Typhoid fever can be presented with profound bleeding manifestation.
• Acute infection can also result in systemic activation of coagulation.

## 1 Introduction

Typhoid fever is an infection that is mainly transmitted orally through food or water contaminated with *Salmonella typhi*. There are an estimated 11–21 million cases of typhoid fever and approximately 128,000–161,000 deaths annually, with the majority of the cases occurring in South Asia, South East Asia, and Sub-Saharan Africa [1]. Common symptoms are fever, with digestive symptoms such as abdominal pain, diarrhea, and bloody stool. Non-specific symptom profile complicates clinical diagnosis, with symptoms that are common to other diseases occurring in typhoid-endemic areas. The mainstay for laboratory confirmation is blood culture, but this has limited sensitivity of approximately 40–60%, due to the window period for detecting organisms circulating in the bloodstream being usually early in the course of the disease and particularly in the first week of the illness, as well as widespread use of antimicrobials [2]. Cases of typhoid fever with disseminated intravascular coagulation (DIC) are rarely reported, even though typhoid fever is endemic in Indonesia [3]. Here, we present a case of a patient with typhoid fever, sepsis and DIC in our low-resource setting. This case was reported in line with the Surgical CAse REport (SCARE) criteria [4].

## 2 Case presentation

A 4-year-old male referral case from a district hospital was experiencing fever, decreased level of conscious, and massive bleeding from his gastrointestinal tract and nose. The patient had been born spontaneously, full term, without history of any underlying disease. None of his parents and siblings have coagulation problems.

Eleven days before admission to Dr. Sardjito Hospital, the patient experienced a fever and cough. The patient was taken to a community health center and assessed with an upper respiratory tract infection. He was treated with acetaminophen and antimicrobial agent, but there was no clinical improvement. Five days before admission, he was referred by a community health center to a district hospital with a suspected diagnosis of dengue fever due to fever and thrombocytopenia, platelet level was 90,000 cells/μL.

Patient was hospitalized in the district hospital for five days, while the fever still persisted. During his hospitalization there was diarrhea, melena, and a hypovolemic shock on the fourth day of admission which was successfully treated with fluid resuscitation. Laboratory examination revealed that there was thrombocytopenia (the lowest level of platelet was 67,000 cells/μL), and leukopenia (2,600 cells/μL). Patient was diagnosed with dengue hemorrhagic fever with unspecified secondary infection. He was treated with antimicrobial agent, acetaminophen, omeprazole, fluid therapy, and platelet transfusion. Antimicrobial therapy that was given included ceftriaxone for 3 days, which was switched to ampicillin-sulbactam on the fourth day of hospitalization, and switched again with ceftazidime on fifth day of hospitalization. Patient had been given 10 L of intravenous fluid using ringer lactate for five days which caused edema. Patient was referred to our hospital, Dr. Sardjito Hospital, due to decrease of consciousness, history of shock and no improvement in clinical symptoms.

The patient's body weight was 22.0 kg, and height 101.0 cm. He was obese based on the weight for height ratio. His general condition was apathetic; his temperature was 37.4 °C. Glasgow Coma Scale was E2 M4 V1, isochoric pupil with normal pupillary light reflex. His heart rate was 160 beats/minutes, blood pressure measured 153/108 mmHg (>95th percentile), good peripheral pulse and capillary refill time less than 2 seconds. Respiratory rate was 64 times/minutes, and oxygen saturation was 87% with rebreathing mask 12 L per minute. His breathing sounds were vesicular and rhonchi in both lungs. Physical examination revealed that his abdomen was distended, with decreasing abdominal sound and ascites. Liver was palpated 9 cm below the right hypochondrium, indicating hepatomegaly. No lymphadenopathy was identified. Massive bleeding was indicated from nasal bleeding, nasogastric tube bleeding, and melena.

Initial laboratory examination found thrombocytopenia (75,000 cells/μL), increase of transaminase enzyme (ALT 4,668 U/L, AST 616 U/L), abnormal blood coagulation (PPT 40.3 s, aPTT 83.8 s, Fibrinogen <7 mg/dL, D-dimer 10,400 ng/mL), lactatemia (lactate 5.42 mg/dL), and increased level of procalcitonin (procalcitonin 11.98 ng/ml) (Table 1). DIC score was 7 from thrombocytopenia. Urinalysis was normal. IgG and IgM anti Dengue were negative. Serology IgM TUBEX®TF test for Salmonella typhi was +8. Pediatric Logistic Organ Dysfunction 2 (PELOD 2) score was 10, and pediatric Sequential Organ Failure Assessment (pSOFA) was 5. Based on clinical and laboratory findings, the patient was diagnosed with typhoid fever, sepsis, and DIC.

**Table 1:** Laboratory examination during hospitalization.

Laboratory Examination	Days of admission										Unit	Reference value
	1	2	3	4	5	6	7	8	10	13		
Leukocyte	7,830	7,140	5,460	3,200	2,280		4,300	4,460	6,710	3,340	cells/μL	4,500–11,500
Lymphocyte	31.3	25.8	31.3	36.6	35.5		20	12.3	11.9	27.2	%	18–42
Neutrophil	62.3	68.4	65.4	59.4	54		60.5	72.5	76.3	67.1	%	50–700
Monocyte	6.4	5.2	2.6	3.1	9.2		18.6	14.1	11.5	5.1	%	0
Eosinophil	0	0	0	0	1.3		0.7	0	0	0	%	0
Basophil	0	0.6	0.7	0.9	0		0.2	1.1	0.3	0.6	%	0
Hemoglobin	7.4	7.1	9.1	8.9	8.7		13.2	12.9	13	11.1	g/dL	12–15
Hematocrite	21	20.3	31.6	26.3	23.9		38	37.1	37.6	32.3	%	35–49
Platelet	75,000	81,000	71,000	48,000	18,000		32,000	25,000	25,000	39,000	cells/μL	150.000–450.000
MCV	75.8	82.5	83.3	82.4	79.9		84.1	84.3	83.4	83	fL	80–94
MCH	26.7	28.9	28.2	27.9	29.1		29.2	29.3	28.8	28.5	pg	26–32
MCHC	35.2	35	33.8	33.8	36.4		34.7	34.8	34.6	34.3	g/dL	32–36
Reticulocyte	0.47										%	0.50–1.50
Procalcitonin	11.98										ng/ml	<0.5
Albumin	2.64					3.23		2.68	2.51	1.93	g/dL	3.97–4.94
BUN	7.5					14.1		12.6	37.7	59.9	mg/dL	6–20
Creatinine	0.4					0.15		0.1	0.21	1.33	mg/Dl	0.5–0.9
Glucose	72		106	109	106	97	98		107		mg/dL	80–140
PPT	40.3		17.5			18.7	21.2	19.1	22.6	42.4	second	12.3–15.3
aPTT	83.8		39.8			46.1	47.6	35.8	47.8	63.1	second	
Fibrinogen	<7		126				143		<8	<8	mg/dL	148–380
D-dimer	10,400		12,840				13,350		12,610	>20,000	ng/mL	<230
Anti-Thrombin 3		31	51	49		60	63				%	80–120
AST	4,668		2,742			1,358		1,089	1,252	2,493	U/L	<56
ALT	616		390			302		308	296	274	U/L	<39
Total bilirubin	4.88					8.97	9.74		11.4	10.68	mg/dL	<1.0
Direct bilirubin	4.13						7.66		9.38	8.9	mg/dL	0.0–0.2
Indirect bilirubin	0.75						2.08		2.02	1.78	mg/dL	0.2–0.8
Sodium	134			138		144				134	mmol/L	132–141
Potassium	4.04			3.33		2.89				3.48	mmol/L	3.5–5.1
Chloride	100			103		91				98	mmol/L	97–107
Calcium	1.88			1.74		1.78				1.25	mmol/L	2.15–2.55
Magnesium	2.26			2.09		1.83				1.71	mg/dL	1.60–2.40
Gamma GT										72	U/L	<87
Phosphatase Alkaline										287	U/L	<409
Ammonia										313.7	μg/dL	27.2–102.0
pH	7.375		7.40		7.43	7.53	7.43		7.42	7.312	mmHg	7.35–7.45
PCO2	32.1		42.8		55.4	43.8	47.2		46.4	33.2	mmHg	35–45
PO2	189		129.9		152.3	187.2	94.1		107.2	102	mmHg	80–95
SO2	100		97.9		98.9	99.5	97.2		97.5	97	%	96–97
BE	−6		2		11.9	13.5	6.8		5.9	−9	mmol/L	−2 - +3
HCO3	18.8		26.7		37.3	37.3	31.6		30.4	16.6	mmol/L	22–26
OI	3,17		2,69		3.6	2.9	4.7		4.1	3.9		
Lactate	5.42		0,99		1.04					5.07	mmol	0.36–1.25
IgG Dengue	Negative											Negative
IgM Dengue	Negative											Negative
TUBEX®TF		+8										Negative
IgG Leptospira									Negative			Negative
IgM Leptospira									Negative			Negative
IgM HAV										Negative		Negative
HbsAg										Negative		Negative

The patient was treated with meropenem 40 mg/kgBW/8 hours, dopamine, omeprazole 1 mg/kgBW/12 hours, sucralfate 250 mg/6 hours, furosemide 1 mg/kgBW/8 hours, tranexamic acid 15 mg/kgBW/8 hours, and acetaminophen. DIC management was given (including fresh frozen plasma, platelet concentrate, packed red cells, and antithrombin 3 factors 500 unit). Meropenem was chosen due to history of ceftriaxone and ceftazidime usage in previous hospital.

The patient was hospitalized in a pediatric intensive care unit for 14 days. During hospitalization, temperature instability, hypotension, and massive bleeding were observed. Laboratory examination during hospitalization showed leukocytosis, persistent thrombocytopenia, hypoalbuminemia, coagulation dysfunction, and abnormal liver function (Table 1). Blood culture was performed four times during hospitalization and the results were negative. Blood culture and fecal culture in the second week of fever using gall culture for typhoid were negative. The patient passed away after 14 days of hospitalization due to multiple organ dysfunction syndrome.

## 3 Discussion

Here, we reported a case of a 4-year-old male patient with typhoid fever and disseminated intravascular coagulation (DIC). Incubation period of Salmonella typhoid is 1–2 weeks. In typical cases, a gradual increase of body temperature, known as “step ladder”, relative bradycardia, and hepatomegaly are common in the first week of onset. Sustained high temperature with apathetic facial expression is observed in the second week of onset. In the third week, intestinal perforation and GIT bleeding are common manifestations. The fourth week is normally the recovery phase. The most common complications due to typhoid infection are hepatitis, bone marrow suppression, and paralytic ileus [5].

The patient's case of typhoid fever was diagnosed using TUBEX®TF with the score being +8, even though blood gall culture test resulted negative in the second week of fever. A positive TUBEX®TF result was defined as a reading of ≥4. TUBEX®TF has 73.0% sensitivity and 69% specificity [6]. Blood culture has limited sensitivity of approximately 40–60%, due to the window for detecting organisms circulating in the bloodstream being usually early in the course of the disease and particularly in the first week of the illness, and the widespread use of antimicrobials [2]. A meta-analysis showed that bone marrow culture has a higher sensitivity rate than blood culture with the sensitivity of 90% [7]. Blood culture in patients after receiving antimicrobials showed less sensitivity than no prior antimicrobial use. Blood volume specimen plays an important role in sensitivity of culture, in which sensitivity increased by 3% (95% CI, 1%–6%) for each additional ml of blood cultured. A 2-mL specimen showed 0.51 (95% CI: 0.44–0.57) and a 10-mL specimen showed 0.65 (95% CI: 0.58–0.70) of sensitivity [7].

Sepsis due to *Salmonella typhoid* is uncommon [8]. Adu-Gyamfi et al. reported a 28 year-old male with Salmonella sepsis in November 2019. The patient came to hospital and presented with septic shock after a ten-day history of abdominal pain, malaise, vomiting, and diarrhea. Initial laboratory examinations found electrolyte imbalance, leukocytosis, thrombocytosis, and increasing C Reactive Protein level. He had an admission APACHE II of 31 and SOFA score of 10. Patient was operated due to rupture appendix suspicious, intraoperative revealed peritoneal inflammation, primarily in the right iliac region, involving a severely inflamed, thickened terminal ilium, dilated colon, and multiple mesenteric lymph nodes with mildly appendix inflammation without perforation or necrosis. The blood and intra-abdominal specimens isolated *S. typhi* which was sensitive to ciprofloxacin and the patient improved with it [9]. Another case reported by Nishida et al. was a 7 year-old boy with typhoid fever complicated by sepsis and DIC. Patient presented with fever, diarrhea, vomiting, excessive drowsiness without any underlying disease. Initial laboratory examinations found thrombocytopenia, liver dysfunction, high level of ferritin, and abnormal coagulation with DIC score of 4. Blood culture isolated *S. typhi*. Patient was treated with ceftriaxone and the patient was clinically improved and discharged in the 14th day of hospitalization [10].

DIC is mostly a subclinical event, and the severe bleeding complications are not typically found in typhoid fever, although DIC score indicates an imbalance of coagulation and fibrinolysis which are markedly elevated in patients with typhoid [11]. Nishida et al. reported a case of typhoid fever complicated by DIC in a 7-year-old male, with acute DIC score of 4, but no sign of bleeding was found [10]. Coagulation problems involve three major processes: pro-coagulation, anti-coagulation, and fibrinolysis. A typhoid patient demonstrates increased plasma prothrombin fragments as well as D-dimer level, prolonged prothrombin time, and lower protein C and anti-thrombin concentrations. Repeated tests of coagulation markers during convalescence showed a return toward normal values [11]. DIC in this patient can be a part of the multi-organ dysfunction due to sepsis syndrome [5].

Acute infection can also result in systemic activation of coagulation. Thrombocytopenia is one of hematological features of typhoid; 18–44.9% of patients with typhoid fever suffer from thrombocytopenia [12]. The mechanism of thrombocytopenia in typhoid patients remains vague. It has been postulated that there are defects in production of platelets due to the direct effect of the toxin produced by Salmonella on the bone marrow, while others have suggested the destruction of non-immune platelets due to DIC [11].

One limitation of our study was the bone marrow culture for typhoid fever was not performed in our patient. Bone marrow culture for typhoid is not a routine protocol in our institution. Bone marrow culture is usually performed if there are negative results of blood and feces culture; unfortunately, our patient passed away before the results of these cultures.

## 4 Conclusions

Sepsis and DIC are rare complications of typhoid fever. Typhoid fever can be presented with profound bleeding manifestations other than gastrointestinal bleeding, since it is a common symptom of typhoid fever. Further research should be conducted to postulate the association between typhoid fever and DIC.

## Ethical Approval

The informed consent form was declared that patient data or samples will be used for educational or research purposes. Our institutional review board also do not provide an ethical approval in the form of case report.

## Sources of funding

The authors declare that this study had no funding source.

## Author contribution

Nurnaningsih, Vincencius William, Desy Rusmawatiningtyas conceived the study and approved the final draft. Nurnaningsih, Vincencius William, Desy Rusmawatiningtyas, Firdian Makrufardi, Intan Fatah Kumara, drafted the manuscript, and critically revised the manuscript for important intellectual content. Nurnaningsih, Vincencius William, Desy Rusmawatiningtyas, Firdian Makrufardi, Intan Fatah Kumara facilitated all project-related tasks.

## Consent

Written informed consent was obtained from the patient's parents for publication of this case report and accompanying images. A copy of the written consent forms is available for review by the Editor-in-Chief of this journal on request.

## Registration of Research Studies

This is not a ‘first in humans’ report, so it is not in need of registration.

## Guarantor

Nurnaningsih.

## Provenance and peer review

Not commissioned, externally peer-reviewed.

## Declaration of competing interest

No potential conflict of interest relevant to this article was reported.
